# Cytokine levels and associations with symptom severity in male and female children with autism spectrum disorder

**DOI:** 10.1186/s13229-017-0176-2

**Published:** 2017-12-02

**Authors:** Anne Masi, Edmond J. Breen, Gail A. Alvares, Nicholas Glozier, Ian B. Hickie, Anna Hunt, Jennie Hui, John Beilby, David Ravine, John Wray, Andrew J. O. Whitehouse, Adam J. Guastella

**Affiliations:** 1 Autism Clinic for Translational Research, Brain and Mind Centre, Central Clinical School, Sydney Medical School, University of Sydney, 100 Mallett Street, Camperdown, New South Wales 2050 Australia; 2 Australian Proteome Analysis Facility, Macquarie University, Sydney, Australia; 3 Telethon Kids Institute, University of Western Australia, Perth, Western Australia Australia; 4 Cooperative Research Centre for Living with Autism (Autism CRC), Long Pocket, Brisbane, Queensland Australia; 5 Pathwest Laboratory Medicine WA, Nedlands, Western Australia Australia; 6 School of Pathology and Laboratory Medicine, University of Western Australia, Perth, Western Australia Australia; 7 School of Population Health, University of Western Australia, Perth, Western Australia Australia; 8 School of Pediatrics and Child Health, University of Western Australia, Perth, Western Australia Australia

**Keywords:** Autism spectrum disorder, Cytokine, Behavior, Pediatric, Severity

## Abstract

**Background:**

Autism spectrum disorders (ASDs) are complex, pervasive, and heterogeneous neurodevelopmental conditions with varying trajectories, significant male bias and largely unknown etiology. However, an understanding of the biological mechanisms driving pathophysiology is evolving. Immune system aberrations, as identified through cytokine profiles, are believed to have a role in ASD. Altered cytokine levels may facilitate identification of ASD subtypes as well as provide biological markers of response to effective treatments. Research exploring the relationship between cytokine profiles and ASD symptoms is, however, in its infancy. The objective of this study was to explore relationships between cytokine levels and the severity of ASD and other clinical traits.

**Methods:**

Multiplex assay techniques were used to measure levels of 27 cytokines in plasma samples from a cohort of 144 children diagnosed with ASD.

**Results:**

Overall, results showed a significant negative association between platelet-derived growth factor (PDGF)-BB, and the severity of ASD symptoms. Furthermore, a significant interaction with sex suggested a different immune profile for females compared to males. ASD symptom severity was negatively associated with levels of 4 cytokines, IL-1β, IL-8, MIP-1β, and VEGF, in females, but not in males.

**Conclusions:**

Results of the present study suggest that an altered cytokine response or profile is associated with the severity of ASD-related symptoms, with sex a potential modifier of this relationship. Further research in larger populations which recognizes the importance of sex comparisons and longitudinal assessments are now required to extend and further describe the role of the immune system in ASD.

**Electronic supplementary material:**

The online version of this article (10.1186/s13229-017-0176-2) contains supplementary material, which is available to authorized users.

## Background

Autism spectrum disorders (ASDs) are complex, pervasive, and heterogeneous neurodevelopmental disorders of primarily unknown etiology and pathogenesis. Recent epidemiological studies estimate prevalence to be as high as 1 in 68 [1]. Diagnosis is currently based on clinical observation of behavioral features, defined as persistent deficits in social communication and interaction, and restricted, repetitive patterns of behavior, interests or activities; severity classifications are based on required levels of support [2]. While ASD is considered a lifelong condition [3], developmental trajectories vary significantly [4] with a range of prognoses [5]. Sex is believed to have an important role in development, with significantly more males diagnosed with ASD (4:1) [6] and different characteristic phenotypes presenting for females [7]. Many genes have been proposed to converge on common pathways affecting neuronal and synaptic homeostasis [8]. Environmental factors may also play a role in etiology through, for example, the impact of maternal immune activation or antibodies on a developing brain or by impacting a susceptible physiology [9–11]. This complex etiology, manifesting as vast clinical heterogeneity, has been a catalyst for investigations aiming to characterize potential biological subtypes of ASD.

Immune system alterations related to immunogenetics, maternal immune activation, and a family history of autoimmune disorders, suggests a relationship between ASD and the immune system [12]. Accumulating evidence of alterations in central and peripheral immune system functioning supports the proposal that there is a subgroup of individuals with ASD who have some form of immune system dysregulation [11]. Previous work has highlighted significantly altered peripheral levels of primarily pro-inflammatory cytokines in ASD compared to healthy controls [13]. Levels of both pro-inflammatory and anti-inflammatory cytokines and chemokines have been associated with the severity of aberrant behavior and more impaired developmental and adaptive function [14–16]. However, numerous differences in immune function have been identified in ASD, suggesting that these aberrations may be due to reduced immune system regulation rather than a dominance of one type of inflammatory response signal [11].

Despite extensive immunological evidence suggesting immune system aberrations in ASD, further research is required to clarify the relationship between immune profiles and ASD symptoms [12]. Such investigations may further knowledge about potentially useful biomarkers for ASD and the severity of symptoms, and provide clues for potential causative relationships and factors that might characterize subgroups of ASD. The pathophysiological significance of commonly identified comorbidities in ASD, such as gastrointestinal dysfunction and sleep disorder, with respect to the identified immune system aberrations, is also unknown [17–19]. While understanding the influence of sex has been a recent research priority [20], partially because of the higher ratio of ASD diagnosis of males [21], to date, there has been limited investigation of potential sex differences in immunological profiles [22]. Sex-specific immunological aberrations have been described in a rodent model, with females exhibiting attenuation of induced behavioral and immunological alterations compared to males [23]. Therefore, there is a need to characterize the relationship between immune system dysfunction, specifically cytokine alterations, and severity of ASD symptoms, including disordered sleep and gastrointestinal dysfunction, between males and females.

The present study utilized a large, well-characterized repository of biological samples and multiplex assay techniques to investigate the relationship between cytokine levels and symptom severity of ASD. Cytokine profiles were investigated in relation to clinical traits, including variability in social functioning, sleep problems, and gastrointestinal dysfunction, along with the role of sex.

## Methods

### Participants

Participants (113 males, 31 females) were part of the Western Australian Autism Biological Registry (WAABR), located at the Telethon Kids Institute in Perth, Western Australia [24]. Ethics approval was granted by the Human Ethics Committee at Princess Margaret Hospital for Children in Perth, Western Australia. Informed consent was provided by the primary caregiver. Participants were recruited during the period January 2011 to March 2013 through newspaper advertisements and via flyers distributed among local service providers and clinicians. Children with a DSM-IV clinical diagnosis of autistic disorder, Asperger’s syndrome, or pervasive developmental disorder—not otherwise specified—were included in the study. Diagnosis in Western Australia mandates a consensus diagnosis following a multidisciplinary assessment by a pediatrician, clinical psychologist, and speech-language pathologist [25]. The Autism Diagnostic Observation Schedule-Generic (ADOS-G) was administered to each participant [26]. The ADOS-G is a semi-structured assessment using simple activities and questions to elicit and observe the communicative, social, and stereotyped behaviors relevant to the diagnosis of ASD. Research-accredited professionals administered the ADOS-G to all participants. Each participant’s ADOS-G raw scores and total score was converted to the updated ADOS-2 calibrated severity score [27–29], as this score is less influenced by child characteristics than raw totals. ADOS diagnostic classification was based on a range of severity scores, where 1 reflects minimal to no evidence of autism spectrum-related symptoms, and 10 represents a high severity of autism spectrum-related symptoms. Based on the level of autism-related symptoms and comparison scores, participants were categorized as mild/moderate or severe, as per established procedures [30]. Calibrated domain scores for social-communication and restricted and repetitive behavior were also derived [31]. A primary caregiver completed a questionnaire about family history, including details of previously diagnosed comorbidities and gastrointestinal problems (classified as present or absent), the Social Responsiveness Scale (SRS) [32], and the Children’s Sleep Habits Questionnaire (CSHQ) [33]. The SRS is a 65-item questionnaire which measures social behavior, language, and repetitive behavior/restricted interests. Higher total score indicates higher severity of social impairment of ASD. The CSHQ is a 33-item questionnaire assessing the frequency of common sleep problems in children with higher scores indicating more disordered sleep. All participants followed the same schedule of assessments, with specimen collection occurring immediately following the ADOS-G assessment.

### Blood sample collection procedure

Peripheral blood (non-fasting) was collected into anticoagulant treated tubes (EDTA, 4 ml vacutainer, K2E, 7.2 mg, BD). The tube was inverted immediately after collection to mix the EDTA with the blood as per standard procedure. Samples were centrifuged for 10 min at 3500 rpm at room temperature in an Eppendorf 5810R centrifuge. The harvested plasma was stored at − 80 ^ο^C, and the analysis was conducted on the day of the first thawing of samples. The samples were defrosted on ice and a 100 μl aliquot was supplied for the multiplex assays.

### Cytokine analysis

Cytokine analysis was conducted using a magnetic bead-based multiplex assay system, which facilitated the detection and quantification of multiple cytokines from a single sample of plasma. A human cytokine 27-plex assay (Bio-Plex Pro Human Cytokine 27-plex #M500KCAFOY) simultaneously quantified levels of 27 cytokines (eotaxin, fibroblast growth factor (FGF) basic, granulocyte colony-stimulating factor (G-CSF), granulocyte–macrophage colony-stimulating factor (GM-CSF), interferon (IFN)-γ, interleukin (IL)-1β, IL1 receptor antagonist (IL-1ra), IL-2, IL-4, IL-5, IL-6, IL-7, IL-8, IL-9, IL-10, IL-12p70, IL-13, IL-15, IL-17, interferon gamma-induced protein (IP)-10, monocyte chemotactic protein (MCP)-1, macrophage inflammatory protein (MIP)-1α, MIP-1β, platelet-derived growth factor (PDGF)-BB, regulated on activation normal T cell expressed and secreted (RANTES), tumor necrosis factor (TNF)-α and vascular endothelial growth factor (VEGF)). All standards, reagents, and samples were prepared according to the manufacturer’s instructions (Bio-Plex® Multiplex System, Bio-Plex Pro™ Human Cytokine, Chemokine and Growth Factor Assays; Bio-Rad Laboratories Pty., Ltd. Hercules, CA, USA). The plates were analyzed using a multiplexing diagnostic instrument (LUMINEX®200™; Luminex Corp.), and fluorescent data for each cytokine was extracted using the manufacturers software (xPONENT for LX200 3.1.971.0; Luminex Corp.). Blood samples for the 144 participants were analyzed blinded to participant’s severity of ASD and all other characteristics including sex. Samples were spread across two plates and two runs (one plate per run). One sample was excluded from cytokine estimations due to misclassification. Standards and blanks were analyzed in duplicate and two duplicate plasma samples were analyzed on each plate to allow an assessment of assay uniformity.

Cytokine level values calculated from participant plasma samples can be lower than the associated lowest cytokine standard or blank. Participant cytokine expression values lower than the associated blanks and standards are considered a result of different matrix effects in plasma and the diluent used in blanks and standards [34] or possibly due to the fact that the actual analytes used with the standards are constructed using recombinant proteins that may differ from natively expressed analytes found in the biological samples [35]. Therefore, a background correction procedure for estimating cytokine levels was used [36, 37]. The correction procedure assumes that any non-specific binding occurring in the blank and standards are nullified by matrix effects in the test samples. Output from the formula was a corrected subject median fluorescence value for each cytokine. Fluorescence values were used as, unlike concentrations, there are no missing values, a level of detection (LOD) does not need to be specified, and low signals can be analyzed which adds more statistical power for testing differences in analyte expression levels. See Additional file 2 for further information. The best matching standard was the standard that had the observed median fluorescence value closest to the median of the subjects for that cytokine. The fluorescence value for the associated best matched standard was used to estimate the expected concentration of that standard using back calculation [38]. Several models were tested to estimate the expected concentrations for the best matched standard for each cytokine (Additional file 1: Table S1). Using correlations between expected and estimated concentration for best matched standards, and associated correlations (*R* ^2^), estimates according to a third order polynomial fit to the log-log transformation of the expected concentrations and fluorescence values yields the best fit standards compared to unweighted 4 parameter logistic (4PL) and 5PL curve fits (Additional file 2: Figure S1). Consequently, this method was used to predict the participant cytokine levels.

As different plates contained different groups of participants, a normalization procedure was developed to address any potential differences in cytokine levels between the two runs. The log2 of fluorescence values were modeled for run and cytokine interaction, taking in to account the level of each cytokine on each plate. The residuals from the model hold the differences in cytokine levels due to any explanatory variable, which was not cofounded with run differences. Additional file 1: Figure S2 illustrates the effect of the run normalization procedure.

### Statistical analysis

Descriptive statistics were calculated for participant characteristics. Missing values were imputed using the multiple imputation by chained equations (MICE) method (Additional file 1: Table S2) [39, 40]. A variable clustering procedure was used to identify homogeneous clusters of variables and eliminate variables conveying similar information [41]. Multinomial logistic regression was undertaken to assess that each plate was balanced with respect to cofactors, or participant characteristics, across runs [42]. All statistical tests were done using R version 3.2.3 (2015-12-10). Type 3 ANOVA tests were done using ANOVA from the car package [43]. Interaction tests were done using test interactions routine from the phia package [44]. Wilcoxon two sample and signed-rank tests were done using the R’s Wilcox test from the coin package [45]. Multiple test corrections were done via the Holm method [46]. Cytokine correlations were obtained using Spearman’s rank correlation. The network visualization of the Spearman’s rank correlation matrix between cytokine levels were obtained using R’s graphing package qgraph version 1.3.3 [47]. qgraph is a visualization tool and a tool for applying network analysis from data for estimating network structures, where the network can be weighted (correlations) or unweighted and undirected. Here, each node represents a cytokine and each edge the correlation between the connecting nodes. For example, qgraph can draw a correlation network, not just as circular network but where strongly correlated nodes are drawn near each other [48]. Generally, correlation networks are drawn fully connected, i.e., showing all non-zero edges between nodes, and when the number of nodes is large this can often make the network hard to interpret. One solution, using model selection to estimate the best graph structure, is to remove edges, set them to zero, with weak correlations using a user specified threshold. Depending on how this is done, the centrality of the network can also be affected. However, thresholding does not consider how removal of these edges changes relationships (correlations) within the network, or how well the network explains the data. An alternative approach and a well-established method for simultaneously estimating parameters and performing model selection on a correlation network, as done here, is to employ LASSO, using the R glasso package [49]. These network models were selected using penalized maximum likelihood solution in which the likelihood is penalized for the sum of absolute parameter estimates using LASSO [50], via qgraph, which can be used to estimate a network that minimizes the Extended Bayesian Information Criterium (EBIC) [51].

## Results

Samples for this analysis were collected from 144 participants, aged between 2 and 18 (mean 8.2 SD ± 4.2). Sex distribution was 78% male and 22% female. Eighty-three percent of participants identified as Caucasian. Additional demographic and clinical characteristics of participants are presented in Table 1. The variable clustering procedure resulted in the inclusion of six variables in the reduced set: age, sex, gastrointestinal symptoms, severity of ASD, sleep score, and SRS total score (Table 1). Assessment of covariate balance with respect to the two different plates or runs identified a run difference only due to sex (*p* = 0.042) (Additional file 1: Table S3).

**Table 1:** Participant clinical characteristics

		Total	Male	Female
*N*		144	113	31
Age (years)				
	Mean	8.16	7.96	9.53
	SD	4.21	4.05	4.79
	Range	2.08–18.25	2.83–18	2.25–18.25
BMI (kg/m2)				
	Mean	18.34	16.74	21.12
	SD	4.63	2.05	7.09
	Range	12.26–46.96	13.50–21.74	13.45–46.96
Head circumference (cm)				
	Mean	53.86	53.67	54.20
	SD	3.01	2.21	3.46
	Range	46–62	49.5–59	46–61
ADOS module				
	Module 1	35	28	7
	Module 2	58	49	9
	Module 3	42	31	11
	Module 4	9	6	3
Severity (ADOS comparison score)				
	Median	6	6	6
	Range	1–10	1–10	1–9
Severity of symptoms				
	Mild/moderate	85	68	17
	Severe	59	45	14
SRS				
	Mean	88.18	86.43	97.27
	SD	15.71	11.09	19.11
	Range	44–126	64–106	44–126
Sleep scores				
	Median	12.5	53	56
	Range	1–31	46–78	46–72
GI symptoms				
	Absent	85	65	20
	Present	59	47	11

*Abbreviations*: *BMI* body mass index, *ADOS* autism diagnostic observation schedule, *SRS* social responsiveness scale, *GI* gastrointestinal

Descriptive statistics for cytokine levels in plasma samples are shown in Table 2. Significance analysis of mixed effect modeling identified an interaction in cytokine levels with respect to two clinical covariates: age (*p* < 0.001) and the severity of ASD (*p* = 0.02; Table 3, Additional file 2: Table S4). There was no interaction between cytokine levels and remaining clinical variables (SRS, sleep, and gastrointestinal symptoms) or a significant main effect. The effect of sex was also investigated to determine if the interaction between cytokine expression and covariates were due to sex differences. Statistically significant effects in females were identified for interactions between cytokine levels and age (*p* = 0.03), severity of presentation (*p* < 0.001), and SRS score (*p* = 0.04), while in males there was a statistically significant effect between cytokine levels and age (*p* < 0.001; Table 3). Significance for the interaction effects with respect to age, severity, and SRS were then investigated for males and females. Reduced levels of IL-1β (*p* < 0.001), IL-8 (*p* < 0.001), MIP-1β (*p* = 0.006), PDGF-BB (*p* = 0.005), and VEGF (*p* = 0.03) were associated with increased severity of autism in females (Table 4). Reduced levels of PDGF-BB (*p* = 0.03) were associated with increased severity of autistic symptomatology in males (Table 4). With the exception of IL-15, cytokine levels appear to decrease with respect to increasing severity (Table 4). Higher levels of IL-8 (*p* = 0.03) were associated with higher scores on the SRS in females. Increased levels of IL-15 (*p* = 0.04) and reduced levels of IL-8 (*p* = 0.005) and MIP-1β (*p* = 0.009) were associated with increasing age in males, while reduced levels of IP-10 (*p* = 0.001) was associated with increasing age in females (Table 4).

**Table 2:** Cytokine median fluorescence values and ranges

Cytokine	Range	Median	IQR
Basic FGF	37–150	71	60–85.5
Eotaxin	35–392	94	67.25–151.5
G-CSF	26–189	57.5	45.5–80.5
GM-CSF	60–1128	406	279.5–550.25
IFN-γ	17–164	43.5	33–61.75
IL-10	31–333.5	77	61–96
IL-12p70	32.5–346	66	48–89.75
IL-13	25.5–598	81	60–133
IL-15	25–427	97	72–130.25
IL-17A	40–222.5	90.5	76.5–105.25
IL-1β	19–541	57	39–96.25
IL-1RA	19–719	52	40–68.5
IL-2	35–563.5	85	70–102
IL-4	20–158	45.5	35–61.25
IL-5	15–136.5	28	22–39
IL-6	26–402.5	48	41–66.5
IL-7	17–157	28.5	24–37
IL-8	26–1716	97	62.25–224.5
IL-9	74–1397	138	113.5–166.75
IP-10	56–2275	371.5	200.25–659.5
MCP-1	41.5–331.5	91	75.25–123
MIP-1α	33–193	75	59.75–93.5
MIP-1β	101.5–2070.5	449	275.5–698.25
PDGF-BB	221–7644.5	1382.5	715.25–2542.75
RANTES	3842–17,915	12,039	8658.5–14,675
TNF-α	19–185	32.5	26–42
VEGF	71.5–941.5	157	110–207.5

IQR (interquartile range). IQR is based on dividing a data set into quartiles Q1, Q2, and Q3 that divides a rank-ordered data set into four equal parts. IQR = Q3-Q1

*Abbreviations*: *Basic FGF* basic fibroblast growth factor, *G-CSF* granulocyte colony-stimulating factor, *GM-CSF* granulocyte macrophage colony-stimulating factor, *IFN-γ* interferon-γ, *IL* interleukin,*IL.1RA* IL-1 receptor antagonist, *IP-10* interferon gamma-inducible protein 10, *MCP-1* monocyte chemotactic protein-1, *MIP-1α* macrophage inflammatory protein-1α, *MIP-1β* macrophage inflammatory protein-1β, *PDG* platelet derived growth factor-BB, *RANTES* regulated on activation, normal T cell expressed and secreted, *TNF-α* tumor necrosis factor-α, *VEGF* vascular endothelial growth factor

**Table 3:** Significance analysis of interaction effects in cytokine levels and clinical covariates

	Combined sex			Males			Females		
Effect	Chisq	Df	Pr(>Chisq)	Chisq	Df	Pr(>Chisq)	Chisq	Df	Pr(>Chisq)
(Intercept)	387.78	1	< 0.001	239.73	1	< 0.001	105.51	1	< 0.001
Severity	3.28	1	0.07	1.48	1	0.22	0.88	1	0.35
Age	0.01	1	0.91	0.12	1	0.73	0.44	1	0.51
SRS	0.13	1	0.72	0.06	1	0.81	1.35	1	0.25
Sleep	0.08	1	0.78	0.23	1	0.63	0.38	1	0.54
GI	0.53	1	0.47	0.96	1	0.33	0.25	1	0.62
Analyte	1079.27	26	< 0.001	787.12	26	< 0.001	247.60	26	< 0.001
Severity:analyte	43.64	26	0.02	16.42	26	0.93	71.10	26	< 0.001
Age:analyte	75.23	26	< 0.001	60.36	26	< 0.001	41.61	26	0.03
SRS:analyte	10.96	26	1.00	19.09	26	0.83	40.28	26	0.04
Sleep:analyte	17.09	26	0.91	12.47	26	0.99	28.20	26	0.35
GI:analyte	9.65	26	1.00	13.11	26	0.98	23.97	26	0.58

Analysis of deviance tables (type III Wald chi-square tests) for the statistical model: mY~(severity + age + sex + SRS + sleep + GI)*Analyte + (1|ID). Where mY represents the normalized log2 of the fluorescence responses with respect to run (Additional file 1: Figure S2)

**Table 4:** Adjusted slope for Age, Severity and SRS

	Age				Severity				SRS			
	Male		Female		Male		Female		Male		Female	
Analyte	Slope	Pr(>Chisq)	Slope	Pr(>Chisq)	Slope	Pr(>Chisq)	Slope	Pr(>Chisq)	Slope	Pr(>Chisq)	Slope	Pr(>Chisq)
Basic FGF	− 0.01	0.89	0.00	0.98	− 0.04	0.39	− 0.06	0.37	0.00	0.97	0.00	0.97
Eotaxin	− 0.03	0.16	− 0.02	0.77	− 0.06	0.13	− 0.11	0.07	0.00	0.97	−0.01	0.85
G-CSF	0.02	0.48	0.04	0.55	−0.03	0.48	− 0.03	0.68	0.01	0.96	0.00	0.97
GM-CSF	− 0.01	0.85	0.02	0.85	−0.01	0.89	− 0.01	0.87	0.01	0.96	0.00	0.98
IFN-γ	− 0.02	0.77	0.00	0.98	− 0.04	0.34	− 0.07	0.35	0.00	0.97	− 0.01	0.97
IL-10	− 0.03	0.16	− 0.02	0.85	− 0.01	0.89	− 0.08	0.22	− 0.01	0.90	− 0.01	0.97
IL-12p70	−0.04	0.07	0.00	0.98	− 0.02	0.68	− 0.12	0.05	−0.01	0.88	0.00	0.97
IL-13	0.01	0.77	− 0.01	0.89	− 0.01	0.75	− 0.05	0.44	0.00	0.97	− 0.01	0.70
IL-15	0.04	0.04*	0.04	0.48	0.00	0.96	0.04	0.54	0.00	0.97	0.00	0.97
IL-17A	− 0.02	0.77	0.00	0.99	− 0.03	0.48	− 0.04	0.54	0.00	0.97	0.00	0.97
IL-1β	− 0.02	0.72	0.02	0.85	−0.06	0.15	− 0.20	< 0.001*	−0.01	0.88	0.01	0.85
IL-1RA	− 0.01	0.85	0.02	0.77	− 0.04	0.35	− 0.08	0.22	0.00	0.97	0.00	0.97
IL-2	0.00	0.98	0.01	0.87	0.00	1.00	−0.04	0.57	0.00	0.97	0.00	0.97
IL-4	0.00	0.98	0.01	0.87	−0.04	0.37	−0.07	0.31	0.00	0.97	0.00	0.97
IL-5	− 0.02	0.77	0.00	0.98	− 0.05	0.22	− 0.06	0.39	0.00	0.97	− 0.01	0.85
IL-6	− 0.01	0.84	0.02	0.85	− 0.04	0.39	− 0.06	0.39	0.00	0.97	0.00	0.97
IL-7	0.00	0.95	0.01	0.98	− 0.04	0.34	− 0.06	0.35	0.00	0.97	0.00	0.97
IL-8	− 0.06	0.01*	0.02	0.77	− 0.05	0.22	− 0.29	< 0.001*	0.00	0.97	0.02	0.03*
IL-9	− 0.01	0.85	0.00	0.98	− 0.01	0.74	0.00	1.00	− 0.01	0.90	0.00	0.97
IP-10	− 0.04	0.09	− 0.10	0.001*	− 0.05	0.22	− 0.02	0.74	− 0.01	0.80	− 0.02	0.21
MCP-1	0.01	0.91	0.02	0.84	− 0.03	0.48	− 0.05	0.48	0.00	0.97	0.00	0.97
MIP-1α	− 0.01	0.84	− 0.01	0.89	− 0.03	0.48	− 0.03	0.60	0.00	0.97	0.00	0.97
MIP-1β	− 0.05	0.01*	− 0.03	0.63	− 0.01	0.75	− 0.16	0.01*	− 0.01	0.85	0.01	0.97
PDGF-BB	− 0.04	0.08	− 0.02	0.85	− 0.08	0.03*	− 0.16	0.01*	−0.01	0.80	0.01	0.85
RANTES	− 0.01	0.88	0.01	0.87	− 0.02	0.54	− 0.09	0.20	0.00	0.97	0.01	0.85
TNF-α	−0.02	0.72	0.00	0.99	− 0.05	0.22	− 0.05	0.44	0.00	0.97	0.00	0.97
VEGF	− 0.04	0.09	0.01	0.98	− 0.03	0.48	− 0.13	0.03*	0.00	0.97	0.00	0.97

Chisq tests: *p* value adjustment method: false discovery rate

*Abbreviations*: *Basic.FGF* basic fibroblast growth factor, *G.CSF* granulocyte colony-stimulating factor, *GM* granulocyte macrophage colony-stimulating factor, *IFN. γ* interferon-γ, *IL* interleukin, *IL.1β* IL-1 beta, IL.1RA, IL-1 receptor antagonist, *IP* interferon gamma-inducible protein 10, *MCP.1* monocyte chemotactic protein-1, *MIP.1α* macrophage inflammatory protein-1α, *MIP.1β* macrophage inflammatory protein-1β, *PDGF.BB* Platelet derived growth factor-BB, *RANTES* regulated on activation, normal T cell expressed and secreted, *TNF. α* tumor necrosis factor-α, *VEGF* vascular endothelial growth factor. * indicates significant difference at *P* < 0.05 or better

**Fig. 1:**
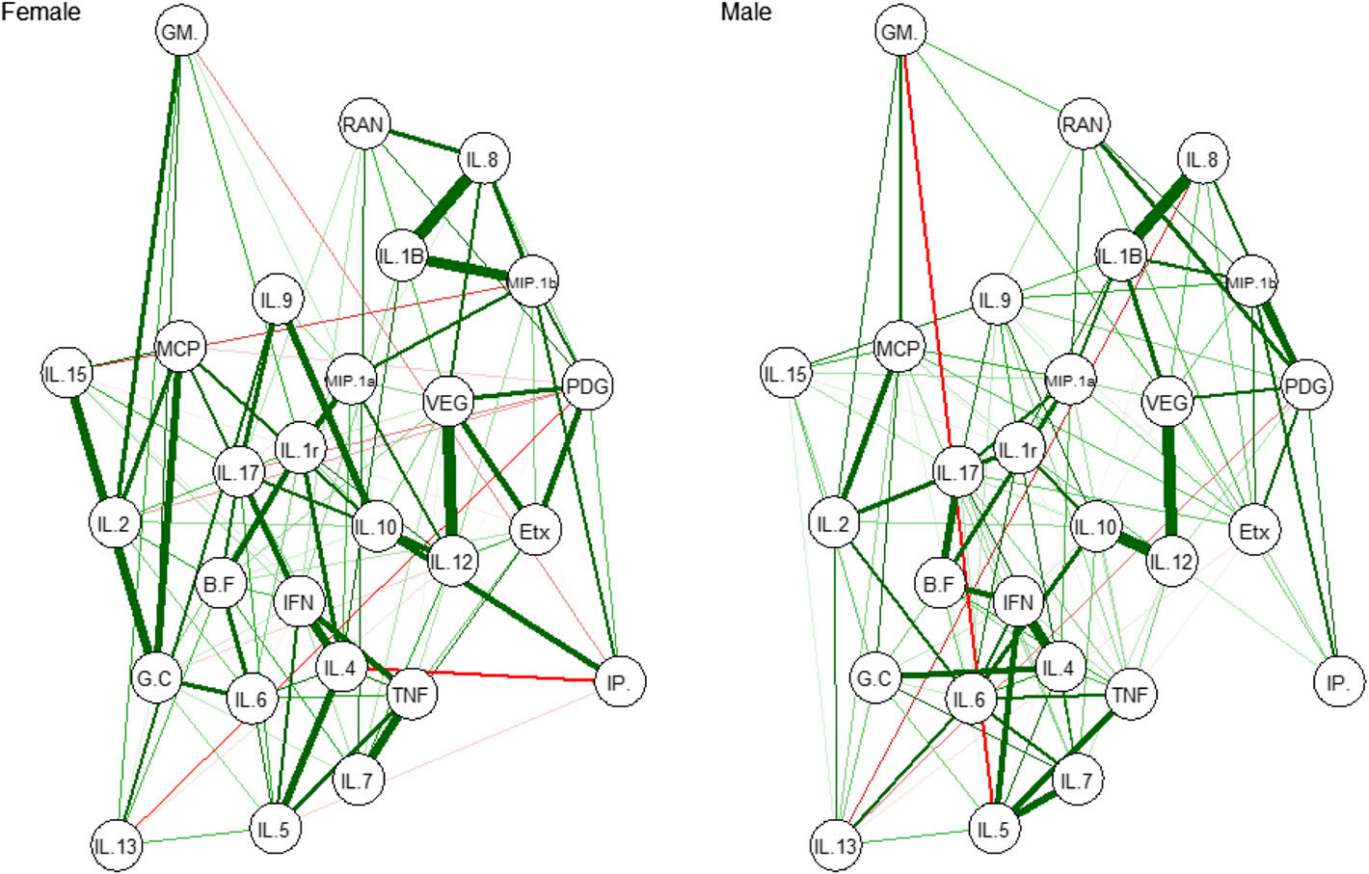
Optimal network graphs representing cytokine correlations separated for sex. Each node represents a cytokine, and each edge represents a Spearman’s correlation between two cytokines. Green edges indicate positive correlations and red edges represent negative correlations. The width of the edge corresponds to the absolute value of the correlation: the higher the correlation, the thicker and more saturated is the edge. Node abbreviations: B.F basic fibroblast growth factor, Etx eotaxin, G.C granulocyte colony-stimulating factor, GM granulocyte macrophage colony-stimulating factor, IFN interferon-gamma, IL interleukin, IL.1B 1 beta, IL.1r IL-1 receptor antagonist, IL.12 IL-12p70, IL.17 IL-17A, IP interferon gamma-inducible protein 10, MCP monocyte chemotactic protein-1, MIP.1a macrophage inflammatory protein-1 alpha, MIP.1b macrophage inflammatory protein-1 beta, PDG platelet-derived growth factor-BB, RAN regulated on activation, normal T cell expressed and secreted, TNF tumor necrosis factor-alpha, VEG vascular endothelial growth factor

Correlations between the levels of each of the 27 cytokines were analyzed (Additional file 1: Figures S3 and S4), with Spearman’s (ρ) correlations above 0.80 highlighted (Additional file 1: Tables S5-S7). A network visualization of the Spearman’s rank correlation matrix between cytokine expression values was created and also optimized for networks separated for sex (Fig. 1, Additional file 1: Figure S5). In the optimal graph networks, visual inspection suggests different network topologies for males and females.

## Discussion

To our knowledge, this study consists of the largest cohort of male and female ASD children to date investigating the relationship between clinical traits of ASD and cytokine levels. Overall, results show evidence of a significant association between decreased cytokine levels and increasing severity of ASD-related symptoms. In particular, decreased levels of the growth factor PDGF-BB were associated with increased severity of ASD-related symptoms. Results also suggested cytokine expression was moderated by the sex of participants. Exploratory analysis showed that decreased levels of an additional four cytokines (IL-1β, IL-8, MIP-1β, and VEGF) were associated with increased severity of presentation in females only. Additionally, increased levels of IL-8 were associated with greater caregiver reported social difficulties. An interaction effect for age was identified across both males and females, although there were different cytokines interacting with age in males and females. Increasing age in males was associated with higher levels of IL-15, and reduced levels of IL-8 and MIP-1β, while increasing age in females was associated with lower levels of IP-10. There were no associations between the other quantitative clinical traits, sleep behavior or gastrointestinal symptoms, and cytokine levels.

Previous studies have reported associations between levels of various cytokines and aberrant behaviors. For example, higher expression of cytokines, including IL-1β, IL-6, IL-8, and IL-12p40 were associated with more impaired stereotyped patterns of behavior [15] while increased levels of the chemokines MCP-1, RANTES, and eotaxin were associated with worsening behavioral symptoms and cognitive and adaptive ability [14]. Further, lower concentration levels of transforming growth factor beta1 (TGFβ1), a regulatory cytokine, has previously been associated with worsening behavior symptoms and lower adaptive behaviors [52]. These findings are in line with a heightened inflammatory state associated with the severity of autism-related symptoms, a cytokine profile similar to that previously identified in participants with ASD compared to healthy controls [13]. In the present study, higher levels of IL-8, which is involved in the recruitment of predominantly neutrophils to settings of tissue inflammation, were associated with higher severity of social impairments in females only. Our remaining findings revealed primarily decreased levels of significant cytokines associated with increased severity of autism-related symptoms, suggesting an altered cytokine profile may be associated with severity of autism-related symptoms. Unlike the possibility of a negative correlation of levels of PDGF-BB with severity of ASD in males and females identified in this study, a positive association between serum levels of PDGF-BB and worsening stereotyped patterns of behavior has previously been identified in a smaller male only cohort [53]. PDGF-BB has been found to be upregulated in healthy children between ages 7 to 17 compared to younger children and adults [54]. PDGF-BB has a complex profile, with PDGF-BB signaling implicated in development and a range of diseases through numerous roles and pathways [55]. Overall, with the exception of IL-15, a pro-inflammatory cytokine considered to be a central factor in the pathogenesis of celiac disease [56], cytokine levels appeared to decrease with respect to increasing symptom severity. However, for most of these cytokines, these differences did not reach significance at the individual cytokine level.

The association of reduced levels of IL-1β, IL-8, MIP-1β, and VEGF, with higher severity levels of ASD in females suggests sex differences in cytokine expression in children with ASD. This was also supported by the exploratory optimal network visualization graphs of cytokine correlations for males and females. Results from recent DNA analysis suggests that females could be more resilient to genetic insults due to carrying more extreme neurodevelopmental-related genetic mutations than males with the same symptoms [57]. Further investigation, particularly with larger female cohorts of ASD that use repeated testing over time, are now required to further support a potential sex-specific biology underlying different cytokine profiles between males and females with ASD. Our results also suggest that age appears to play an important moderating role in these effects; in males, an overall decline in cytokine levels was associated with increasing age. However, this effect was more mixed in females with only about half of the cytokine levels declining with older age. The physiological changes occurring during the onset of puberty and beyond may contribute to the variations of cytokine levels with age and sex. Age-related changes in cytokine expression in healthy children have been described elsewhere [54, 58, 59], although there is limited age-matched reference data of cytokine expression that can assist with more specific interpretation of these findings.

The results of the present study suggest that an altered cytokine response could be associated with severity of presentation, particularly in females. These results do not corroborate with some previous work that has identified associations between increased levels of pro-inflammatory cytokines and behavioral impairments in ASD, and are also not consistent with peripheral immune system abnormalities in ASD manifesting only as a pro-inflammatory bias [60]. The elucidation of possible neural circuitry mechanisms that are impacted by an aberrant peripheral immune system resulting in downstream changes in behavior are imperative. Evidence from investigations involving a rodent model deficient in adaptive immunity suggest that social deficits may result from impaired circuitry homeostasis derived from dysfunctional immunity [61], supporting the importance of this type of research in ASDs. The heterogeneity in ASD substantially increases the complexity associated with the identification of either markers of a pathological state that manifest in a subgroup of ASD or biomarkers of response to treatment that are specific and sensitive enough to be consistent measures of change.

The objective of this study was to utilize a well characterized cohort to establish if there is a relationship between cytokine levels and symptom severity of ASD and other clinical traits, including variability in social functioning, sleep problems, and gastrointestinal dysfunction. The levels of cytokines were analyzed using fluorescent data. There is a need for consensus in the literature about the use of absolute or fluorescent levels, and the treatment of concentrations obtained below the sensitivity LOD when presenting cytokine data. The use of absolute values has been more common in past autism research [14, 15]; however, the use of fluorescent levels is also an acceptable method [36]. While the concurrent analysis of a control group would have highlighted any cytokine differences between the ASD cohort and healthy controls, these differences have previously been well established [13] and would not facilitate the identification of potential relationships between the immune system aberrations and severity in ASD. The elucidation of these latter relationships have greater potential to contribute to knowledge about the relationship between symptoms and immune function in autism. Control groups typically vary widely on many characteristics beyond autism symptoms and their use in understanding the immune circuitry underpinning autism profiles may be debated. This cohort reflects the ratio of approximately four affected males to one affected female in the general population; however, larger cohorts of females are required to replicate and extend the sex-related findings. Furthermore, the duplication of samples across plates would have clarified the run difference that was linked to sex. The assessment of the severity of ASD-related symptoms undertaken using the ADOS was conducted by one assessor, strengthening the reliability of diagnoses and evaluations of severity. Additionally, ADOS assessments were conducted on the day of blood draw, therefore cytokine expression levels were relevant to that time point. While the ADOS-2 calibrated severity metric was approximated using a large sample [27], it is not representative of a population distribution of ADOS scores. Diagnostic and assessment measures, such as the ADOS, used in the ASD population are primarily behaviorally defined. There is a growing body of evidence implicating comorbid psychopathology and medical conditions in ASD [62–64], highlighting the importance of comprehensive characterization of participants, particularly with respect to the assessment and reporting of all medical and mental health comorbidities. It is possible that there were participants with an undiagnosed comorbidity, allergies or concurrent illness at the time of blood draw that may elevate risk of immune system dysfunction and peripheral upregulation or downregulation of cytokines. Additionally, describing the onset of symptoms is important as previous research has suggested that regression may be associated with elevated cytokine expression [15].

## Conclusions

The present study highlights the importance of an interdisciplinary approach to address the complexity of an autism presentation [65], particularly with respect to the pathological significance of immune system abnormalities. Longitudinal investigations in large cohorts with comprehensive, clinically relevant comprehensive biological data relating to medical comorbidities found in ASD, such as sleep disorders, and gastrointestinal and immune dysfunction, and controlling for variables that continually interact with the immune system, such as age, pubertal status, infections, and allergies, are required in order to thoroughly characterize the mechanisms of action behind these types of results. Further work is required to extend these findings to determine whether cytokines can be employed as either a sensitive marker of a pathological state-related to a subgroup in ASD or as a biomarker of response to treatment.

## Additional files

Additional file 1: Supplementary Information. (DOCX 3801 kb)

Additional file 2: Quality and level of detection. (DOCX 728 kb)

## Abbreviations

ADOS: Autism diagnostic observation schedule
ADOS-G: ADOS-Generic
ASD: Autism spectrum disorder
BMI: Body mass index
CSHQ: Children’s Sleep Habits Questionnaire
DSM: Diagnostic and Statistical Manual of Mental Disorders
EBIC: Extended Bayesian Information Criterium
FGF: Fibroblast growth factor
G-CSF: Granulocyte colony-stimulating factor
GI: Gastrointestinal
GM-CSF: Granulocyte–macrophage colony-stimulating factor
IFN: Interferon
IL: Interleukin
IL-1ra: IL1 receptor antagonist
IP: Interferon gamma induced protein
IQR: Interquartile range
LOD: Level of detection
MCP: Monocyte chemotactic protein
MICE: Multiple imputation by chained equations
MIP: Macrophage inflammatory protein
PDGF: Platelet-derived growth factor
PL: Parameter logistic
RANTES: Regulated on activation normal T cell expressed and secreted
SRS: Social Responsiveness Scale
TNF: Tumor necrosis factor
VEGF: Vascular endothelial growth factor
