# Supplementary material for: Cytokine levels and associations with symptom severity in male and female children with autism spectrum disorder
Source: Mol Autism. 2017 Dec 2;8:63. doi: 10.1186/s13229-017-0176-2 (PMC5712192; doi:10.1186/s13229-017-0176-2)
Supplement: Supplementary file 1 — Supplementary Information. (DOCX 3801 kb) [file 13229_2017_176_MOESM1_ESM.docx]

Supplementary Information

Table S1. Cytokine concentrations and best matched standard. Concentration is pg/ml.

| **Cytokine** | **Subject Median**  **Fl*** | **Best Matched Standard** | **Best Matched**  **Std Median Fl*** | **Expected Conc**  **[pg/ml]** | **Conc 4PL**  **[pg/ml]** | **Conc 5PL**  **[pg/ml]** | **Conc Poly3**  **[pg/ml]** | **Estimated Sample**  **Concentration [pg/ml]** |
| --- | --- | --- | --- | --- | --- | --- | --- | --- |
| Basic FGF | 71 | S5 | 117.75 | 55.605 | 314.565 | 579.67 | 40.301 | 28.531 |
| Eotaxin | 94 | S6 | 67.25 | 27.527 | 46.969 | 26.463 | 24.43 | 30.345 |
| G-CSF | 58 | S7 | 23 | 12.676 | NA | NA | 18.542 | 35.123 |
| GM-CSF | 406 | S5 | 270.5 | 51.363 | 43.334 | 44.727 | 30.367 | 62.25 |
| IFN-γ | 43.5 | S6 | 19.5 | 33.56 | NA | NA | 31.244 | 74.261 |
| IL-10 | 78 | S7 | 31.75 | 8.577 | 7.395 | 4.406 | 9.525 | 18.068 |
| IL-12p70 | 66 | S7 | 32 | 9.318 | 9.206 | 9.384 | 12.376 | 20.104 |
| IL-13 | 81 | S6 | 51 | 6.813 | 7.136 | 6.762 | 5.034 | 7.813 |
| IL-15 | 97 | S6 | 131.5 | 23.501 | 17.154 | 17.044 | 14.157 | 14.976 |
| IL-17A | 91 | S6 | 63.5 | 24.63 | NA | NA | 17.32 | 31.998 |
| IL-1β | 58 | S7 | 40.25 | 1.887 | 1.626 | 1.601 | 1.636 | 2.091 |
| IL-1RA | 53.5 | S6 | 32.5 | 75.673 | 86.122 | 76.7 | 74.023 | 116.636 |
| IL-2 | 85 | S7 | 50.25 | 4.748 | 15.974 | 15.916 | 13.18 | 9.412 |
| IL-4 | 46 | S7 | 27.5 | 1.013 | 0.909 | 1.056 | 0.778 | 1.051 |
| IL-5 | 28 | S7 | 16 | 5.073 | NA | NA | 5.648 | 7.865 |
| IL-6 | 49 | S7 | 56.75 | 6.528 | 6.707 | 7.624 | 6.043 | 5.454 |
| IL-7 | 28.5 | S7 | 21.25 | 4.1 | NA | NA | 4.197 | 5.09 |
| IL-8 | 97 | S7 | 66.25 | 9.59 | 9.611 | 8.965 | 7.995 | 10.604 |
| IL-9 | 138 | S6 | 186 | 19.909 | 32.58 | 33.402 | 18.503 | 14.913 |
| IP-10 | 369 | S4 | 204.75 | 327.906 | 295.02 | 352.207 | 313.13 | 470.069 |
| MCP-1 | 91 | S6 | 79.5 | 26.77 | 26.048 | 26.177 | 21.637 | 25.138 |
| MIP-1α | 75 | S6 | 53.25 | 2.331 | 1.711 | 1.532 | 1.57 | 2.018 |
| MIP-1β | 449 | S5 | 563.25 | 25.188 | 25.542 | 25.762 | 18.347 | 14.658 |
| PDGF-BB | 1402 | S3 | 2082.5 | 1099.313 | 1067.41 | 1095.845 | 1174.973 | 716.129 |
| RANTES | 12109 | S2 | 12683.5 | 3956 | 7404.633 | 7309.471 | 6204.608 | 5728.855 |
| TNF-α | 32.5 | S7 | 14 | 14.84 | NA | 13.828 | 17.618 | 36.719 |
| VEGF | 157 | S6 | 198.75 | 35.022 | 41.174 | 38.334 | 28.95 | 23.837 |

Abbreviations: Basic FGF, Basic fibroblast growth factor; G-CSF, granulocyte colony-stimulating factor; GM, granulocyte macrophage colony-stimulating factor; IFN-g, interferon-γ; IL, interleukin; IL-1B, IL-1 beta, IL-1RA, IL-1 receptor antagonist; IP, interferon gamma-inducible protein 10; MCP-1, monocyte chemotactic protein-1; MIP-1a, macrophage inflammatory protein-1α; MIP-1b, macrophage inflammatory protein-1β; PDGF-BB, Platelet derived growth factor-BB; RANTES, regulated on activation, normal t cell expressed and secreted; TNF-a, tumor necrosis factor-α; VEGF, vascular endothelial growth factor.

Figure S1.


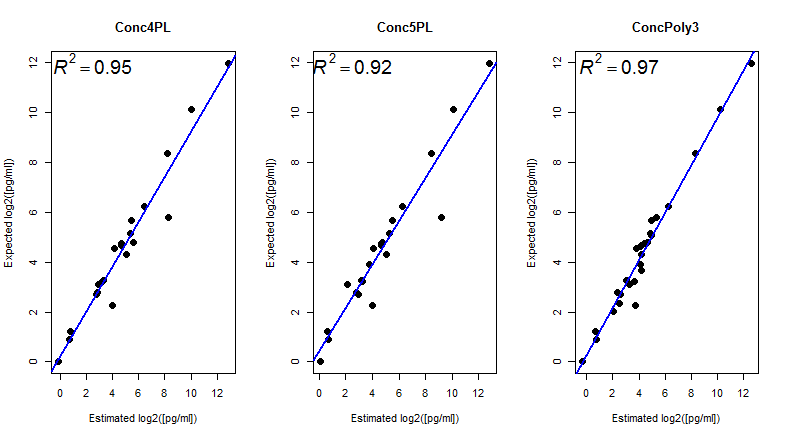


Figure S2. Effect for run normalization procedure.


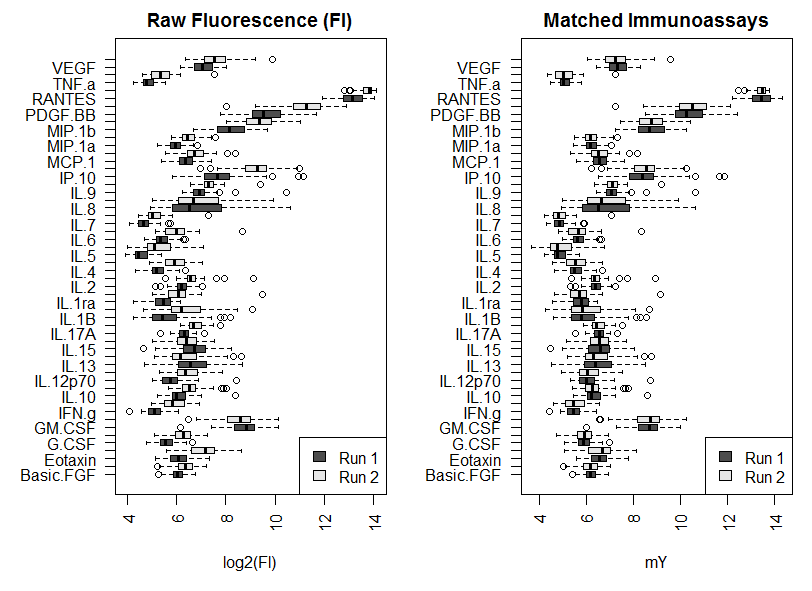


Table S2. Meta data descriptive statistics before and after imputation using multiple imputation by chained equations (MICE) method.

| **Before Imputation** | | | | | | |
| --- | --- | --- | --- | --- | --- | --- |
|  | **Age** | **ADOS**  **Comparison score** | | **SRS** | **Sleep** |  |
| **N** | 144 | 144 | | 135 | 126 |  |
| **min** | 2.08 | 1 | | 44 | 1 |  |
| **max** | 18.25 | 10 | | 126 | 31 |  |
| **range** | 16.17 | 9 | | 82 | 30 |  |
| **median** | 7.33 | 6 | | 88 | 12 |  |
| **mean** | 8.16 | 5.93 | | 87.68 | 13.52 |  |
| **SE.mean** | 0.35 | 0.18 | | 1.34 | 0.62 |  |
| **CI.mean** | 0.69 | 0.36 | | 2.65 | 1.23 |  |
| **var** | 17.72 | 4.9 | | 242.41 | 49.05 |  |
| **std.dev** | 4.21 | 2.21 | | 15.57 | 7 |  |
| **coef.var** | 0.52 | 0.37 | | 0.18 | 0.52 |  |
| **After Imputation** | | | | | | |
|  | **Age** | **ADOS**  **Comparison score** | **SRS** | | **Sleep** |  |
| **N** | 144 | 144 | 144 | | 144 |  |
| **min** | 2.08 | 1 | 44 | | 1 |  |
| **max** | 18.25 | 10 | 126 | | 31 |  |
| **range** | 16.17 | 9 | 82 | | 30 |  |
| **median** | 7.33 | 6 | 88.5 | | 12.5 |  |
| **mean** | 8.16 | 5.93 | 88.18 | | 13.75 |  |
| **SE.mean** | 0.35 | 0.18 | 1.31 | | 0.6 |  |
| **CI.mean** | 0.69 | 0.36 | 2.59 | | 1.18 |  |
| **var** | 17.72 | 4.9 | 246.95 | | 51.35 |  |
| **std.dev** | 4.21 | 2.21 | 15.71 | | 7.17 |  |
| **coef.var** | 0.52 | 0.37 | 0.18 | | 0.52 |  |

| **GI** | Before imputation | After imputation |
| --- | --- | --- |
| Absent | 79 | 85 |
| Present | 51 | 59 |

Table S3. Multinomial test for covariate balance across run 1 and run 2.

| Analysis of Deviance Table (Type II tests) | | |
| --- | --- | --- |
| Response: Plate | | |
| Effect | LR Chisq | Pr (>Chisq) |
| Age | 0.877 | 0.349 |
| **Gender** | **4.116** | **0.042** |
| Severity | 0.266 | 0.606 |
| SRS | 0.212 | 0.646 |
| Sleep | 2.242 | 0.134 |
| GI | 0.020 | 0.887 |

Blood samples for 144 patients were spread across two plates, with one run per plate, representing two runs (run 1 and run 2). Multinomial regression ^1^ where the run was modelled as function of Age + Gender + Severity+SRS+Sleep, followed by ANOVA significance.

Table S4. 3-Way Factorial Analyses for Severity, SRS and Age with respect to Gender and Analyte.

| **A** | **Severity** | | |
| --- | --- | --- | --- |
| **Effect** | **Chisq** | **Df** | **Pr(>Chisq)** |
| (Intercept) | 1382.355 | 1 | 0 |
| Analyte | 3966.233 | 26 | 0 |
| Gender | 0.01 | 1 | 0.921 |
| Severity | 1.866 | 1 | 0.172 |
| Analyte:Gender | 43.115 | 26 | 0.019 |
| Analyte:Severity | 20.759 | 26 | 0.754 |
| Gender:Severity | 0.107 | 1 | 0.743 |
| Analyte:Gender:Severity | 47.204 | 26 | 0.007 |

| **B** | **SRS** | | |
| --- | --- | --- | --- |
| **Effect** | **Chisq** | **Df** | **Pr(>Chisq)** |
| (Intercept) | 253.782 | 1 | 0 |
| Analyte | 926.731 | 26 | 0 |
| Gender | 0.477 | 1 | 0.49 |
| SRS | 0.041 | 1 | 0.84 |
| Analyte:Gender | 50.168 | 26 | 0.003 |
| Analyte:SRS | 23.205 | 26 | 0.621 |
| Gender:SRS | 0.621 | 1 | 0.431 |
| Analyte:Gender:SRS | 50.129 | 26 | 0.003 |

| **C** | **Age** | | |
| --- | --- | --- | --- |
| **Effect** | **Chisq** | **Df** | **Pr(>Chisq)** |
| (Intercept) | 2330.266 | 1 | 0 |
| Analyte | 7687.233 | 26 | 0 |
| Gender | 0.165 | 1 | 0.685 |
| Age | 0.159 | 1 | 0.69 |
| Analyte:Gender | 27.232 | 26 | 0.397 |
| Analyte:Age | 81.595 | 26 | 0 |
| Gender:Age | 0.074 | 1 | 0.785 |
| Analyte:Gender:Age | 27.207 | 26 | 0.399 |

Three mixed effect models were used, one for each parameter Severity, SRS and Age. In each model and for each parameter the other parameters were used as random factors. For example, the severity parameter model was: mY~Analyte*Gender*Severity + (1|ID) + (1|Age) + (1|SRS).

Figure S3. Correlations between cytokines (Spearman ρ).


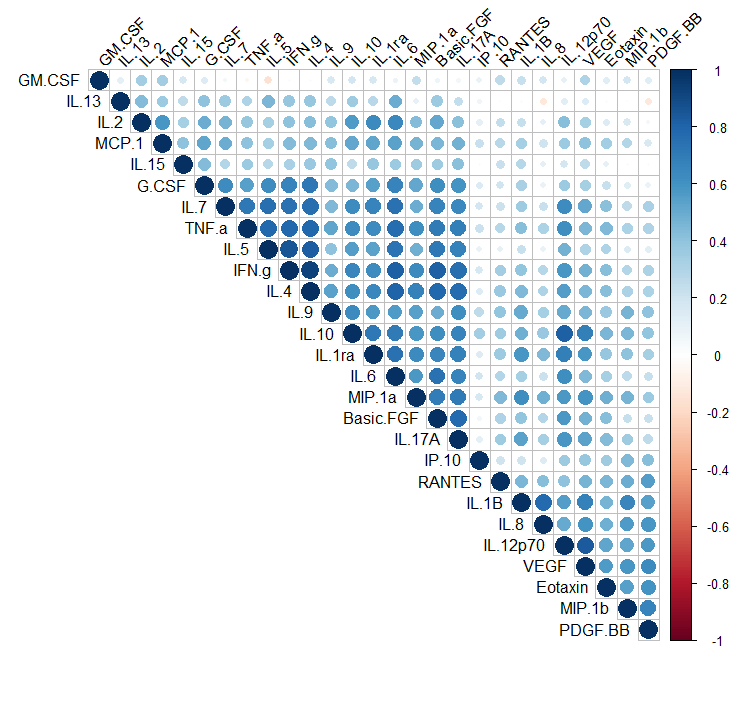


Figure S4. Correlations between cytokines for females (A) and males (B) (Spearman ρ).

**A**

**B**


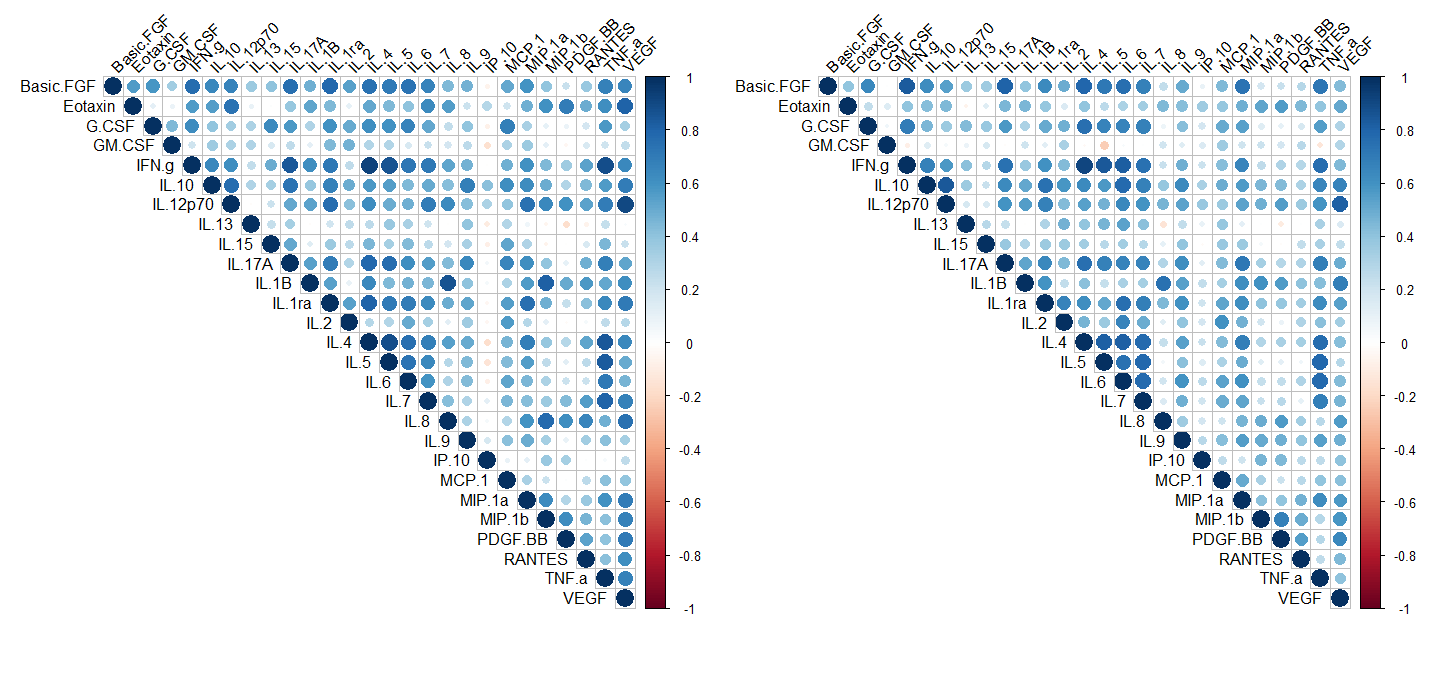


Table S5. Correlations between cytokines overall (Spearman ρ).


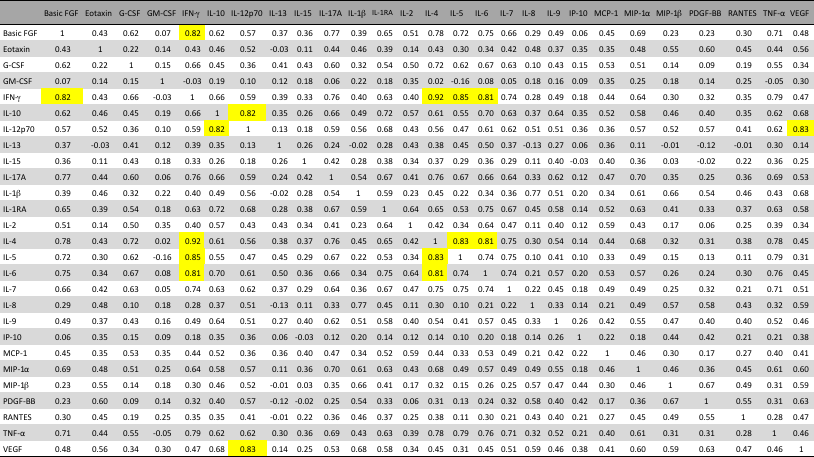


Table S6. Correlations between cytokines for females (Spearman ρ).


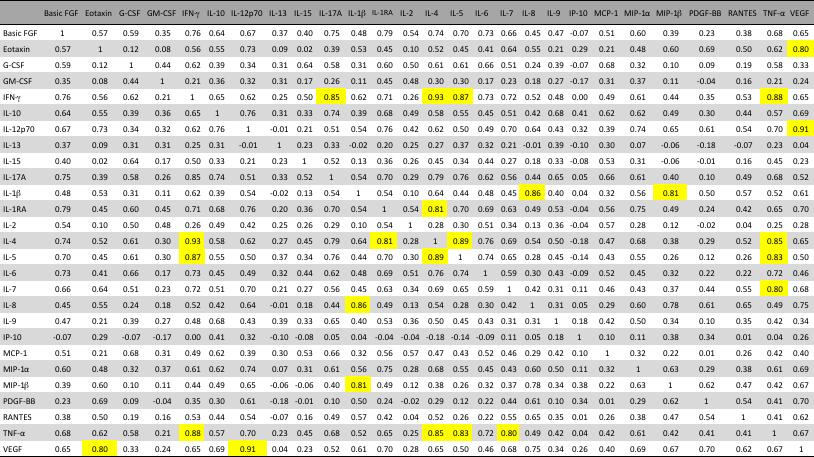


Table S7. Correlations between cytokines for males (Spearman ρ).


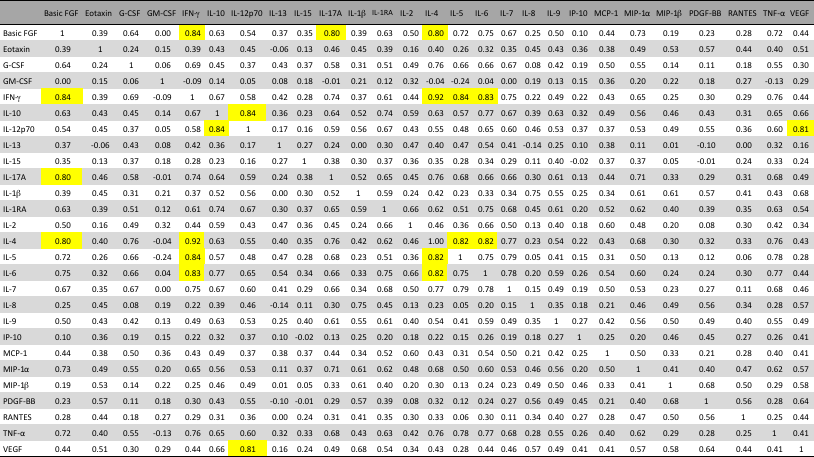


Figure S5. Visualization network of cytokine correlations.


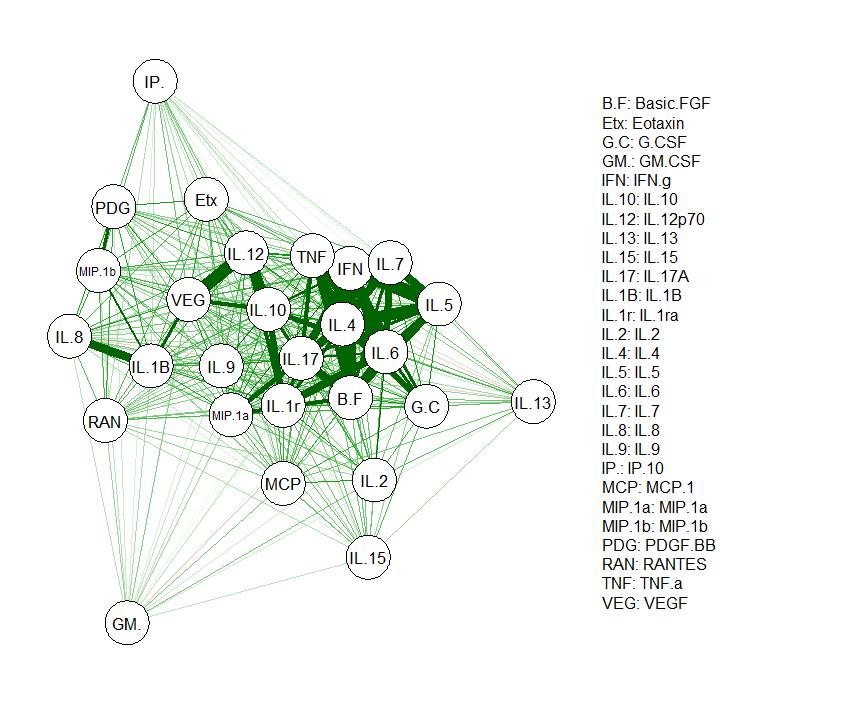


Each node represents a cytokine and each edge represents a Spearman’s correlation between two cytokines. Green edges indicate positive correlations, and the width of the edge corresponds to the absolute value of the correlation: the higher the correlation, the thicker and more saturated is the edge. Abbreviations: B.F, Basic fibroblast growth factor; Etx, Eotaxin; G.C, granulocyte colony-stimulating factor; GM, granulocyte macrophage colony-stimulating factor; IFN, interferon-γ; IL, interleukin; IL.12, IL-12p70; IL.17, IL-17A; IL.1B, 1 beta, IL.1r, IL-1 receptor antagonist; IP, interferon gamma-inducible protein 10; MCP, monocyte chemotactic protein-1; MIP-1a, macrophage inflammatory protein-1α; MIP-1b, macrophage inflammatory protein-1β; PDG, Platelet derived growth factor-BB; RAN, regulated on activation, normal t cell expressed and secreted; TNF, tumor necrosis factor-α; VEG, vascular endothelial growth factor.

**References**

1. Venables WN, Ripley BD (2002): *Modern Applied Statistics with S*. Springer, New York.
