# Supplementary material for: Cytokine levels and associations with symptom severity in male and female children with autism spectrum disorder
Source: Mol Autism. 2017 Dec 2;8:63. doi: 10.1186/s13229-017-0176-2 (PMC5712192; doi:10.1186/s13229-017-0176-2)
Supplement: Supplementary file 2 — Quality and level of detection. (DOCX 728 kb) [file 13229_2017_176_MOESM2_ESM.docx]

# Quality and level of detection

This analysis is performed only on the data associated with Plate 2, which contained 77 of the test samples.

The quality of the sample values associated with plate 2, can be ascertained by plotting the response of the sample distributions (X) for each cytokine against their respective standards and blanks and as seen in Figure 1. The red horizontal line in each plot represents the level of detection for each cytokine as determined by the blank’s mean + 2 times the blank’s standard deviation.


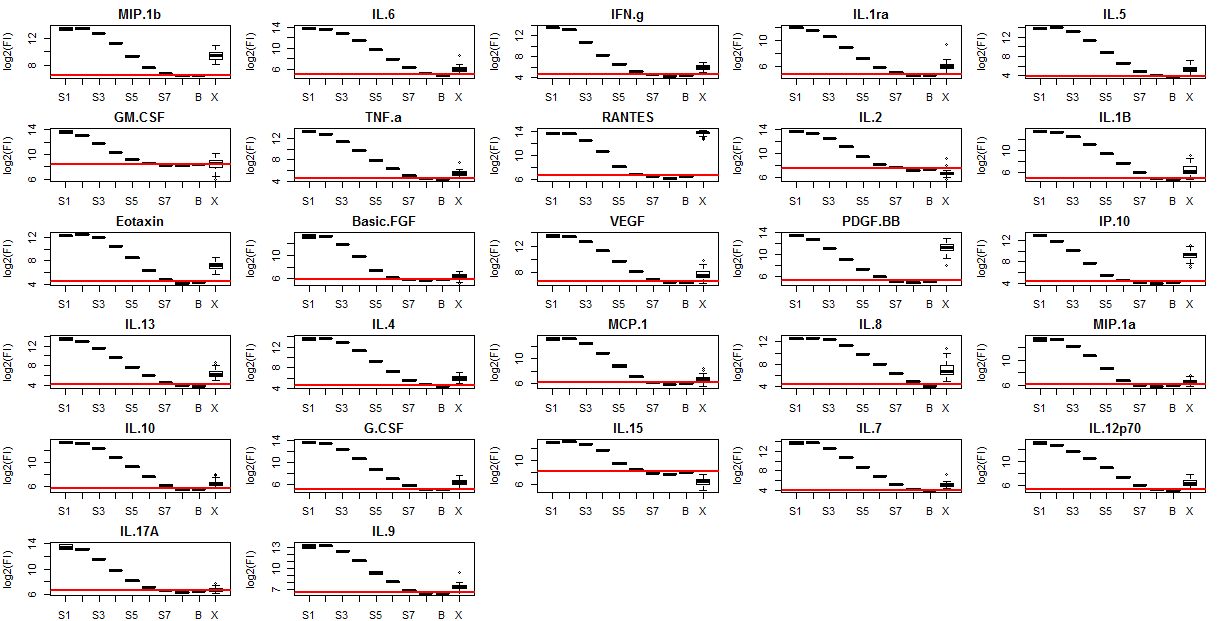


Figure 1: Cytokine log2 sample box plot distributions compared to standards and blanks. Horizontal red line on each plot represents the observed level of detection (LOD) for each cytokine. S1 to S8 represents standards 1 to 8, B represents blank, and X represents the samples for each cytokine.

There are several plots shown in Figure 1 where the level of detection (LOD) is seen to intersect or lie above the median of the sample distributions, for example as seen for IL-2, IL-17A and IL-15. Therefore, it is of interest to see if the distributions for such plots are considered normally distributed or right skewed indicating that the test-sample values (X) are hitting a lower plateau near or below their associated LOD.

The normality of distribution is often reviewed by producing kernel density plots as seen in Figure 2, where the vertical red line represents each cytokine’s LOD. One way to determine the normality of these distributions is to determine their skewness (D.N. Joanes and C. A. Gill, 1988). For example, the skewness of a sample of size 77 (sample size on plate 2) from a random distribution is expected 99.7% (+/- 3 * $\sigma)$of the time to lie between -0.78 and 0.78 as determined via simulation (Figure 3).


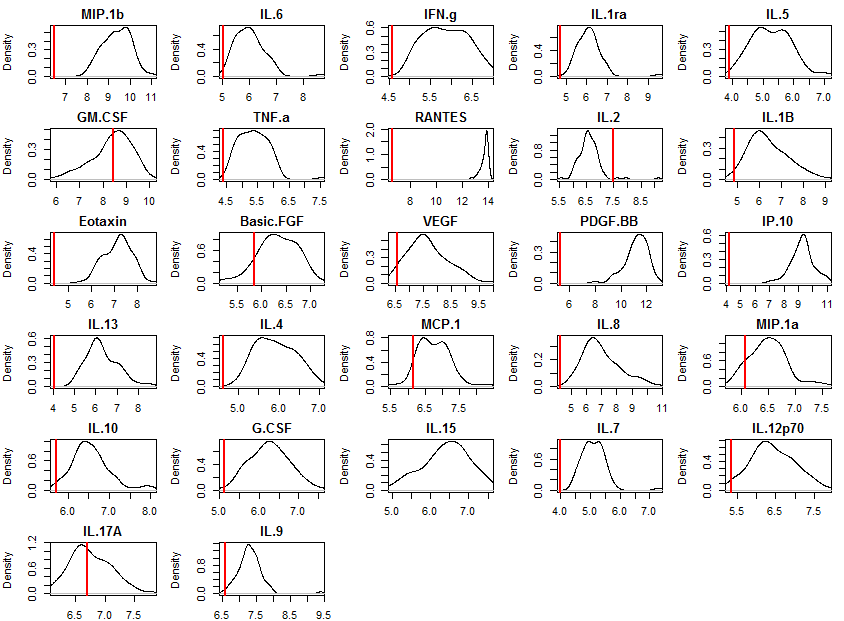


Figure 2: Test-sample cytokine kernel density plots. X-axis represents the log2 of the fluorescence values. The vertical red line represents the respective level of detection (LOD).


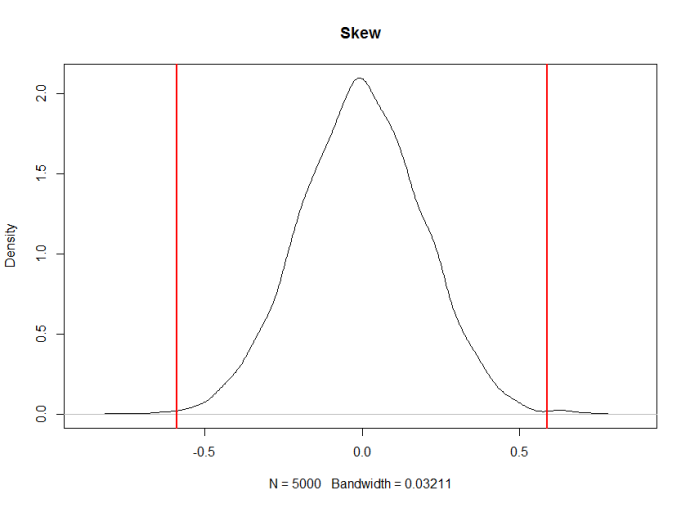


Figure 3. Density of plot of the skewness values determined from 5000 samples of size 77 drawn from a random distribution (mean 0 and standard deviation of 1). Vertical red lines represent the 99.7% confidence limits, $\boldsymbol{\sigma=0.26}$.

Table 1 provides summary statistics for the blanks (B) associated with each cytokine and their level of detection plus the median test sample fluorescence values and skewness for the log2 distribution of the associated test-sample values. Table 1 shows that the distributions for IL-15 and IL-17A are considered normally distributed and not right skewed as expected as they lie close or below their LOD. However, IL-2 is right-skewed. It appears the measured LOD of detection has little to do with the observed quality of data. One reason for this is the matrix used in the immunoassay kit controls (standards and blanks) is not the same as used for the test samples. Therefore, it can be argued there is no real relationship between the control samples and the test-samples.

.

| Cytokine | mean | std | LOD | X.median | X.skew | Normal | Right.skew |
| --- | --- | --- | --- | --- | --- | --- | --- |
| RANTES | 91.25 | 3.889 | 99.028 | 14591.5 | -1.340 |  |  |
| PDGF.BB | 33.5 | 3.536 | 40.571 | 2532.5 | -0.874 |  |  |
| GM.CSF | 345.75 | 0.354 | 346.457 | 370 | -0.634 | YES |  |
| Basic.FGF | 55 | 1.414 | 57.828 | 81 | -0.348 | YES |  |
| IL.15 | 249 | 12.728 | 274.456 | 86 | -0.316 | YES |  |
| Eotaxin | 19.25 | 1.061 | 21.371 | 151 | -0.176 | YES |  |
| IP.10 | 17.5 | 0.707 | 18.914 | 645 | -0.147 | YES |  |
| MIP.1b | 85.75 | 1.768 | 89.286 | 668.5 | -0.043 | YES |  |
| IFN.g | 21 | 1.414 | 23.828 | 57.5 | 0.105 | YES |  |
| G.CSF | 31.25 | 1.768 | 34.786 | 77 | 0.141 | YES |  |
| IL.12p70 | 38.5 | 0.707 | 39.914 | 82.5 | 0.201 | YES |  |
| IL.4 | 21.25 | 1.768 | 24.786 | 59 | 0.214 | YES |  |
| IL.5 | 15 | 0.000 | 15.000 | 38 | 0.299 | YES |  |
| IL.17A | 94.5 | 4.950 | 104.399 | 103.5 | 0.457 | YES |  |
| MCP.1 | 69 | 1.414 | 71.828 | 105 | 0.543 | YES |  |
| MIP.1a | 62 | 2.828 | 67.657 | 88 | 0.547 | YES |  |
| VEGF | 85.5 | 4.243 | 93.985 | 186 | 0.554 | YES |  |
| IL.1B | 26 | 1.414 | 28.828 | 75 | 0.628 | YES |  |
| IL.13 | 14 | 1.414 | 16.828 | 71.5 | 0.720 | YES |  |
| IL.8 | 17 | 1.414 | 19.828 | 109 | 0.802 |  | YES |
| IL.10 | 48 | 2.121 | 52.243 | 90 | 0.905 |  | YES |
| TNF.a | 19.5 | 0.707 | 20.914 | 41 | 0.990 |  | YES |
| IL.6 | 27.75 | 2.475 | 32.700 | 62.5 | 1.420 |  | YES |
| IL.7 | 16 | 0.000 | 16.000 | 33.5 | 1.744 |  | YES |
| IL.9 | 88.75 | 3.889 | 96.528 | 155 | 1.976 |  | YES |
| IL.1ra | 25.5 | 0.707 | 26.914 | 67 | 2.128 |  | YES |
| IL.2 | 162 | 8.485 | 178.971 | 95 | 2.345 |  | YES |

Table 1. Level of detection (LOD) associated with each cytokine expression level. Mean represents the mean fluorescence values of each blank, std represents the observed standard deviation for each blank, X.median represents the test-sample median fluorescence value. X.skew gives the skewness of the observed log2 distribution of the test-sample fluorescence values according to Joanes and C. A. Gill (1998). Normal indicates whether or not each test-sample’s fluorescence log2 values can be considered normal. Like-wise Right.skew is used to indicate which of these distributions are considered to skewed to the right.

## References

D. N. Joanes and C. A. Gill (1998), Comparing measures of sample skewness and kurtosis. The Statistician, **47**, 183–189.
